# Case Report and Literature Review of a Pathologic Mandibular Fracture from Metastatic Esophageal Adenocarcinoma

**DOI:** 10.1155/2018/7860384

**Published:** 2018-12-23

**Authors:** Daniel C. O'Brien, Garrett Jones, Maggie Yell, Jason McChesney

**Affiliations:** ^1^Department of Otolaryngology Head and Neck Surgery, University of West Virginia, Morgantown, WV, USA; ^2^Department of Medical Education, University of West Virginia, Morgantown, WV, USA; ^3^Department of Pathology, Anatomy, and Laboratory Medicine, University of West Virginia, Morgantown, WV, USA

## Abstract

Distant metastasis to the jaws, including the maxilla or mandible, is very rare. Although the mandible is the preferred sight of these rare metastases, it is extremely rare for oral cavity malignancies to present as pathological mandibular fractures. Here, we present a case of esophageal adenocarcinoma metastasizing to the mandible, and the first reported case presenting with a pathological mandibular fracture secondary to esophageal adenocarcinoma. We also review the 9 other reported cases of metastatic esophageal adenocarcinoma to either the maxilla or mandible.

## 1. Introduction

Pathological mandibular fractures are rare and account for only 2% of all mandibular fractures. Causes of pathological mandibular fractures have been reported following tooth removal, dental implant placement, secondary to benign and malignant mandibular pathology, osteomyelitis, osteoradionecrosis, bisphosphonate-related osteonecrosis, and Gorham's disease [1]. Even though only 1% of all oral cavity malignancies are metastatic in origin, when these metastases arise, they are the first sign of the primary disease about 25% of the time [2]. Pathological mandibular fractures caused by metastatic disease often present at a late stage. Secondary to advanced stage at presentation, treatment generally consists of chemotherapy or radiation therapy as a means of palliation. If the tumor is resectable, the gold standard of therapy is primary radical surgery [1].

This case report was reviewed by the University of West Virginia Institutional Review Board and found to be exempt and did not require full IRB review.

## 2. Case Report

A 69-year-old Caucasian male with a past medical history of hypertension, oromandibular dystonia treated with Botox, and recent diagnosis of gastroesophageal reflux disease (GERD) presented to his primary care provider noting an area of his left chin that was numb. The area was small and could be covered with 1 finger. He followed up acutely three weeks later with significant dysphagia for solids, but not liquids. He was urgently referred for an upper endoscopy. Upper endoscopy revealed LA Class D esophagitis with ulceration in the distal esophagus, and biopsies showed inflamed glandular mucosa with at least high-grade dysplasia. These findings were consistent with a diagnosis of Barrett's esophagus secondary to GERD.

**Figure 1:**
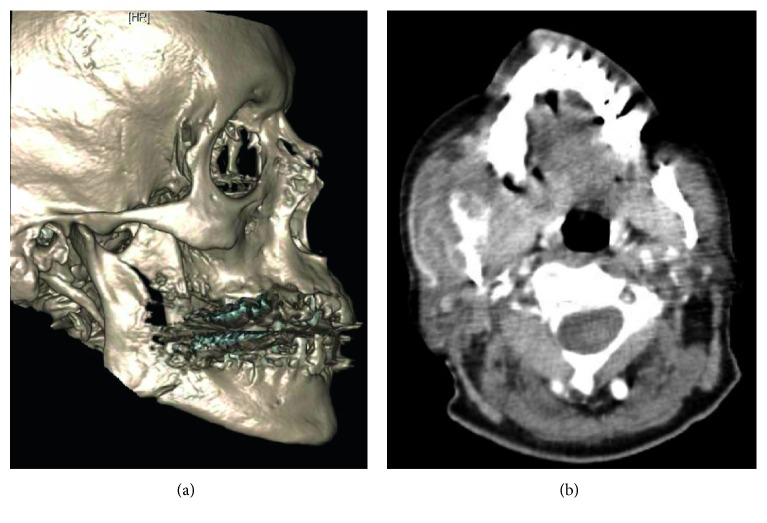
(a) Three-dimensional reconstruction of patient's pathologic fracture. Note the minimally displaced right mandibular angle fracture and right coronoid fracture. (b) Axial soft tissue of the mandible, displaying the mass of the right retromolar trigone.

**Figure 2:**
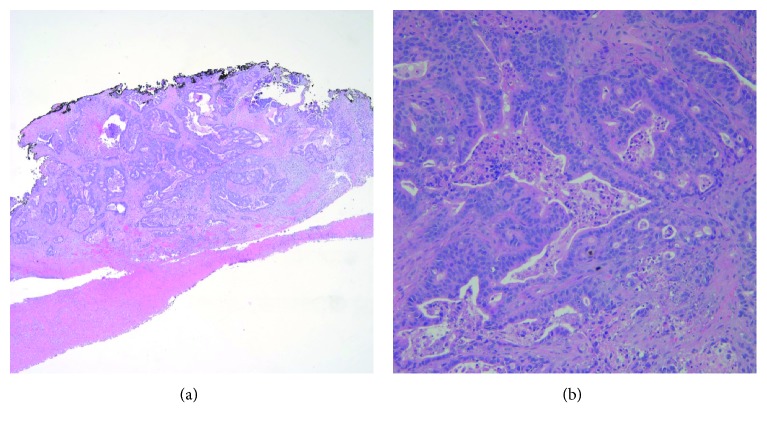
Pathologic specimen of the retromolar trigone biopsy. 2A: 100x magnification of hematoxylin and eosin stain (low power). Back to back gland formation with undermining of squamous mucosa. 2B: 200x magnification hematoxylin and eosin stain (high power). Infiltrating malignant glands containing necrotic debris.

Three weeks after his upper endoscopy, he presented to the emergency department with right jaw pain and swelling after hitting his jaw on a work bench. A CT revealed right mandibular angle fracture and coronoid fracture (Figure 1). The facial trauma team was consulted and, secondary to his oromandibular dystonia, he was discharged on a liquid diet with Augmentin, Peridex, and close follow-up. He was seen in the clinic a week later and denied trismus, malocclusion, or difficulty with his liquid diet. On examination, he was found to have an exophytic mass of the right retromolar trigone, which he noted his teeth had been hitting. This mass was present before his fracture and had gotten larger over time. This mass was biopsied in the clinic and came back as likely metastatic adenocarcinoma (Figure 2).

**Figure 3:**
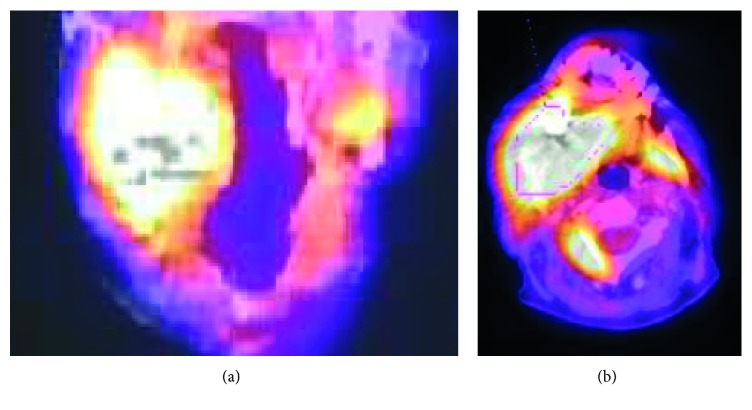
PET/CT of the patient's mandibular mass. This image shows an avidly hypermetabolic mass of the right mandibular angle. (a) Coronal cuts; (b) axial cuts.

The patient underwent a second upper endoscopy. Biopsy taken during this second endoscopy was consistent with a moderately differentiated adenocarcinoma of the distal esophagus. A PET/CT revealed a large, hypermetabolic distal esophageal mass consistent with the given diagnosis of esophageal adenocarcinoma. Hypermetabolic lesions involving the regional lymph nodes, lungs, spine, and right mandible, as shown in Figure 3, were found on PET/CT. These findings were consistent with a Stage IV, TX, NX, M1, and G2, esophageal adenocarcinoma. At that time, he denied smoking, alcohol, illicit drugs, and/or exposure to radiation and carcinogenic chemicals.

The hypermetabolic area of the right mandible, in conjunction with the previous retromolar trigone biopsy, confirmed a likely pathological fracture of the mandible secondary to metastatic esophageal adenocarcinoma. The advanced stage of the cancer made him a poor candidate for surgical intervention for either primary tumor or mandibular metastasis. He was referred for palliative chemoradiotherapy. He passed away one month after diagnosis.

## 3. Discussion

We are reporting a case of a 69-year-old Caucasian male sustaining a pathological mandibular fracture from metastatic esophageal adenocarcinoma. His pathological mandibular fracture occurred 1 week prior to his diagnosis of esophageal adenocarcinoma. There have been 9 previous cases of metastatic esophageal adenocarcinoma to the jaws reported in the literature and are listed in Table 1. None of these cases were associated with a pathological fracture of the mandible or maxilla [3–11]. The only reported case of metastatic esophageal carcinoma causing a pathologic fracture was secondary to an esophageal squamous cell carcinoma. In this previous case, the primary tumor was diagnosed prior to the pathological mandibular fracture [12].

Only 1% of all oral cavity malignancies arise from metastatic disease, and metastasis to the jaw bones is more common than oral soft tissues [2]. Metastasis to the jaws are thought to be by hematogenous spread, with the posterior portion of the mandible, molar, premolar, and angle of the ramus being the most common sites. Metastasis settles in these areas most commonly secondary to the red bone marrow located in these subsites [2, 13]. Tooth extraction is commonly associated with metastasis to the oral cavity. In some cases, metastatic disease causes loosening of the teeth necessitating extraction, and in other cases, extraction of the teeth provides the inflammatory environment favorable to attract cancer cells. The growth factors provided by the bone marrow stromal cells and gingival inflammation are thought to provide a nurturing environment for cancer cells to thrive [2, 13].

In a review of 453 cases of metastatic disease to the jaw bones, the male-to-female ratio was reported as between 1.2 and 1 with a mean age of 53.4 years old at diagnosis [13]. The most common origin for metastasis to the jaw include lung, kidney, liver, and prostate in men and breast, female genital organs, kidney, and colon in women. Metastatic tumors to the oral cavity most commonly present as exophytic lesions. The survival rate of patients with oral metastasis is reported to be 7 months secondary to the severity and progression associated with metastasis [2]. Secondary to significant disease burden, treatment is generally limited to chemotherapy or radiation therapy as a means of palliation, however, radical resection, if possible, has been described [14]. Classic symptoms of metastatic disease to the jaws include rapid, progressive swelling, pain, and paresthesia. Numb chin syndrome or mental nerve neuropathy is characteristic of metastasis to the mandible and includes anesthesia and paresthesia over the chin, lower lip, and submental area. It is worth noting that, in this case, the paresthesia was of the opposite chin from the side of mandibular metastasis. Metastatic disease to the oral cavity should be considered in any patient with symptoms of numb chin syndrome, pain, swelling, or an exophytic lesion [2].

Esophageal adenocarcinoma has become increasingly more common over the last several decades, and it is now more common than esophageal squamous cell carcinoma in the United States. Men have approximately 6-fold higher risk of developing esophageal adenocarcinoma as compared to women [14]. Our patient's only risk factor for developing esophageal adenocarcinoma was being an elderly Caucasian male with GERD. Other risk factors for developing esophageal adenocarcinoma include central adiposity and tobacco consumption, while NSAIDs and H *pylori* are thought to be protective. Alcohol is not considered a risk factor [14]. In recent years, males >65 years old have seen the greatest increase in incidence of esophageal adenocarcinoma [15].

## 4. Conclusion

We report a case of esophageal adenocarcinoma presenting as a pathological mandibular fracture. Review of the literature shows this is the 10^th^ reported case of esophageal adenocarcinoma metastasizing to either the mandible or maxilla and the first reported case of esophageal adenocarcinoma to present as a pathological mandibular fracture.

## Figures and Tables

**Table 1:** Reported cases of esophageal adenocarcinoma to the maxilla or mandible.

	Authors	Year published	Location of metastasis	Age	Sex	Time between primary diagnosis and metastasis
1	Fontaine et al. [3]	1961	Right mandible	55	Male	Concurrent
2	Tideman et al. [4]	1986	Left posterior maxilla	59	Male	4 weeks
3	Jones [5]	1989	Left mandibular body	54	Male	Concurrent
4	Anderson and Peepler [6]	1990	Left mandibular body and ramus	61	Male	Concurrent
5	Willard et al. [7]	2002	Left posterior maxilla	41	Male	9 months
6	Sanchez-Jimenez et al. [8]	2005	Upper left maxilla	63	Male	4 months
7	Tamiolakis et al. [9]	2007	Mandible	NA	NA	NA
8	Jham and Salama. [10]	2011	Mandible	67	Female	NA
9	Lawes et al. [11]	2013	Left mandible	69	Male	7 years
10	O'Brien et al. (This Case)	2018	Right mandibular angle	69	Male	Metastasis identified prior to primary
